# QTL Mapping in Eggplant Reveals Clusters of Yield-Related Loci and Orthology with the Tomato Genome

**DOI:** 10.1371/journal.pone.0089499

**Published:** 2014-02-21

**Authors:** Ezio Portis, Lorenzo Barchi, Laura Toppino, Sergio Lanteri, Nazzareno Acciarri, Nazzareno Felicioni, Fabio Fusari, Valeria Barbierato, Fabio Cericola, Giampiero Valè, Giuseppe Leonardo Rotino

**Affiliations:** 1 DISAFA - Plant Genetics and Breeding, University of Torino, Grugliasco, Torino, Italy; 2 Consiglio per la Ricerca e Sperimentazione in Agricoltura - CRA-ORL, Research Unit for Vegetable Crops, Montanaso Lombardo, Lodi, Italy; 3 Consiglio per la Ricerca e Sperimentazione in Agricoltura - CRA-ORA, Research Unit for Vegetable Crops, Monsampolo del Tronto, Ascoli Piceno, Italy; 4 Consiglio per la Ricerca e Sperimentazione in Agricoltura - CRA-GPG, Genomic Research Centre, Fiorenzuola d’Arda, Piacenza, Italy; 5 Consiglio per la Ricerca e Sperimentazione in Agricoltura - CRA-RIS, Rice Research Unit, Vercelli, Italy; Leibniz Institute of Plant Biochemistry, Germany

## Abstract

In spite of its widespread cultivation and nutritional and economic importance, the eggplant (*Solanum melongena* L.) genome has not been extensively explored. A lack of knowledge of the patterns of inheritance of key agronomic traits has hindered the exploitation of marker technologies to accelerate its genetic improvement. An already established F_2_ intraspecific population of eggplant bred from the cross ‘305E40’ x ‘67/3’ was phenotyped for 20 agronomically relevant traits at two sites. Up to seven quantitative trait loci (QTL) per trait were identified and the percentage of the phenotypic variance (PV) explained per QTL ranged from 4 to 93%. Not all the QTL were detectable at both sites, but for each trait at least one major QTL (PV explained ≥10%) was identified. Although no detectable QTL x environment interaction was found, some QTL identified were location-specific. Many of the fruit-related QTL clustered within specific chromosomal regions, reflecting either linkage and/or pleiotropy. Evidence for putative tomato orthologous QTL/genes was obtained for several of the eggplant QTL. Information regarding the inheritance of key agronomic traits was obtained. Some of the QTL, along with their respective linked markers, may be useful in the context of marker-assisted breeding.

## Introduction

The eggplant (*Solanum melongena* L.) belongs to the Solanaceae family and it is cultivated worldwide, particularly in China (about 60% of world production) and India (about 25%). After potato and tomato, it represents the third most important solanaceous crop species [1], but unlike the former two, it is an Old World (India and China) rather than a New World domesticate [2], [3].

The inheritance of agronomic traits has been intensively studied in the solanaceous crops, and a growing number of genes and quantitative trait loci (QTL) have been identified and even isolated [4]–[14]. Much of this effort has been focused on tomato, potato and sweet *Capsicum* pepper, leaving the eggplant knowledge base rather limited. In a survey of trait inheritance in eggplant, Chadha [15] identified the expected mixture of major genes and polygenes, while Nunome et al. [16] were able to map a number of fruit trait QTL. As an interspecific F_2_ population was the platform for the mapping of some breeding trait QTL [17], [18], the relevance of these loci for intraspecific improvement is probably rather limited. Miyatake et al. [19] were able to define two QTL underpinning parthenocarpy by mapping in an intraspecific population, while strain-specific wilt (*Ralstonia solanacearum*) resistance was shown by Lebeau et al. [20] to be conditioned by a single dominant gene and QTL which are located in two linkage groups. A densely populated intraspecific RAD-tag derived marker based genetic linkage map has recently been used to characterize the genetic basis of traits associated with anthocyanin content [21].

In this paper, we describe the phenotyping, with respect to 20 yield, fruit and morphological traits, of a previously genotyped mapping population bred from a cross between a doubled haploid derivative of the interspecific somatic hybrid *S. aethiopicum* gr. *gilo*(+)*S. melongena* [22] and ‘67/3’, an F_8_ selection from an intra-specific cross in *S. melongena* [23]. The intention was to locate relevant QTL and to explore the possibility of using known syntenic relationships between the eggplant and the tomato genome to infer potential candidate genes underlying some of the major QTL identified.

## Results

### Phenotypic Variation and Inter-trait Correlations

**Figure 1:**
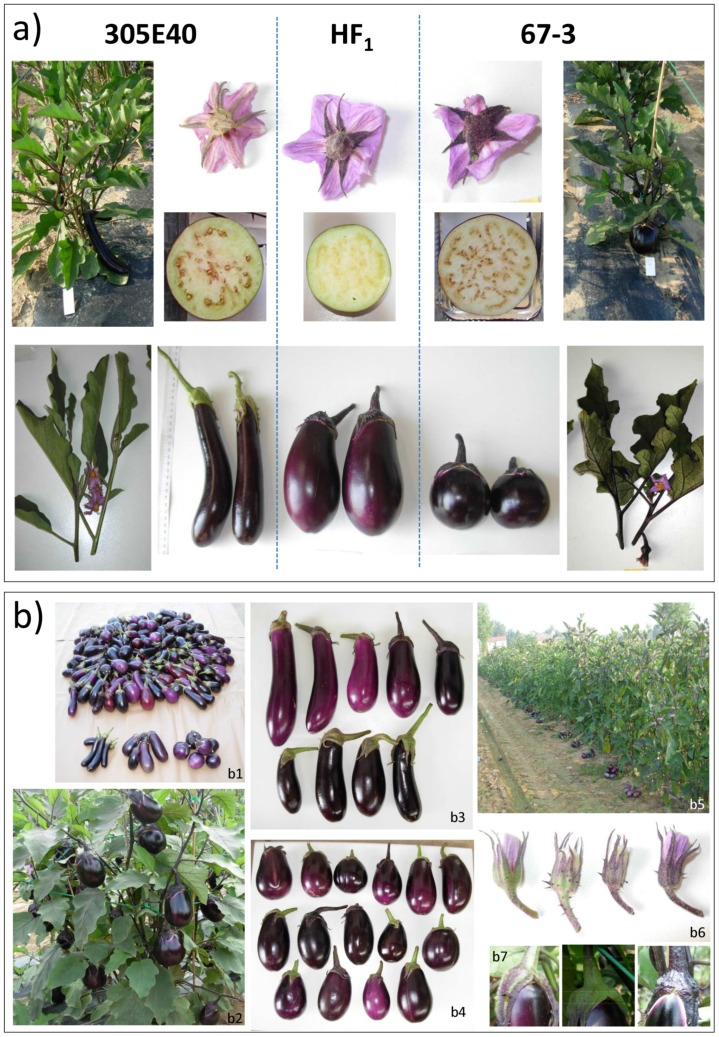
Parental and F2 progeny phenotypes for some of the traits considered. a) Morphological features of the parental lines 305E40 and 67/3, and of the hybrid F1. For each parental lines are shown: whole plant where the different growth habit can be appreciated, a detail of the principal apical shoot with leaves and flowers (where also multiple inflorescence in 305E40 and single flower/inflorescence in 67/3 can be noted), fruits (where colour, dimensions, peduncle length and prickles can be noted), a section of the fruit (where the green ring and number of seed locules can be distinguished) and a flower (presence/absence of prickles). For the hybrid, flower, fruit and fruit section are shown. b) Some morphological features of the segregating F2 progenies. b1: an overview of fruit diversity among the entire progeny with respect to the parental lines (from the left: 305E40, HF1, 67/3); b2: detail of an F2 plant during the harvesting season: the abundance of fruits of this genotype overcome the parental lines (one of the transgressive genotype for yield); b3 and b4: detail of fruit produced by different F2 plants showing variability for colour, dimensions, shape, calyx prickles and peduncle length roughly grouped according to the long (b3) and oval (b4) typologies; b5: a view of F2 field-grown plants after an harvesting; b6: flowers differing for colour and presence of prickles; b7: example of some fruit calyxes differing for colour and presence of prickles.

Trait codes, their performance and broad sense heritability are presented in Table 1. The parental lines contrasted for most of the traits at both sites (Table 1). Compared to ‘67/3’ plants, ‘305E40’ plants set longer, narrower and lighter fruits, which developed on a longer peduncle and formed fewer seed locules and a green ring in the flesh next to the skin; its habit was upright and a higher number of flowers were formed per inflorescence, both the flower calyx and the leaves were prickly (Figure 1). Despite the lower number of fruits produced per plant, the total and early yield of ‘67/3’ was higher than the equivalents in ‘305E40’. The first flush of fruit in both parental lines was larger than the fruit produced later. At both sites, the F_1_ hybrid was intermediate for almost all the traits (Table 1), and F_1_ performance was significantly superior to the better performing parent only with respect to ty and ey in ML (data not shown). In the F_2_ generation, transgressive segregation (as calculated from the raw phenotypic data) with respect to ‘67/3’ was observed in ML for ty (99 plants), tyfn (two plants), ey (21 plants), eyfn (two plants), outfir (seven plants) and fcpri (seven plants) and, with respect to ‘305E40’ were observed for ty (four plants), tyfn (125 plants), tyfw (two plant), ey (four plants), eyfn (40 plants), pedl (three plants) and slon (two plants). In MT, Transgressive phenotypes were found for ty (three plants), tyfn (95 plants), tyfw (17 plants), eyfn (66 plants), fw (seven plants), outfir (four plants), and hab (two plants) towards 305E40 and for ty (43 plants), tyfn (one plant), ey (29 plants), eyfw (two plants ), slon (one plant) towards 67/3 parent (a rough estimation about the number of transgressive individuals can be deduced from Figure S1). The broad sense heritability values were generally higher at ML than at MT. The range was from 0.18 (ey at MT) to 0.98 (gring at both locations) (Table 1). Significant inter-trait correlations (p<0.05) were detected both within and across sites (Table 2). In both ML and MT, production traits (fw, fl, fd1/2, fd3/4, fdmax, tyfn, ty, tyfw, eyfn, ey and eyfw) were uniformly positively correlated with one another, while fs was negatively correlated with fruit weight and diameters. The correlations across sites ranged from +0.285 for slon to +0.897 for fw.

**Table 1:** List of the traits and their units of measurement, mapping population means, standard deviations (SD), coefficients of variation (cv) and broad sense heritabilities.

Trait	Code	Env	Parents means ± SD			F1	F_2_ population	cv	Skewness	SE	Kurtosis	SE	Heritability
			305E40	67/3			mean ± SD						
Total yield (gr)	ty	ML	3088±494.01	5325.25±1113.38	*	8166.75±777.78	7912.61±2783.32	0.35	−0.24	0.19	−0.06	0.39	0.84
		MT	2624.25±600.71	3783±783.7	*	4342.25±460.49	4389.52±1561.29	0.36	0.24	0.19	−0.43	0.39	0.42
Total yield fruit number	tyfn	ML	22.75±4.65	16.25±2.62	*	29.25±2.75	41.61±11.89	0.29	−0.47	0.19	−0.14	0.39	0.84
		MT	21.25±3.5	15.75±3.09	*	23.5±2.64	31.63±9.78	0.31	0.27	0.19	−0.10	0.39	0.49
Total yield av. fruit weight (gr)	tyfw	ML	137.1±14.76	326.02±20.16	*	279.55±15.16	185.90±32.07	0.17	0.18	0.19	−0.06	0.39	0.91
		MT	122.5±10.33	240±11.77	*	185.02±7.10	134.23±24.51	0.18	0.71	0.19	1.98	0.39	0.54
Early yield (gr)	ey	ML	1769.5±444	2743.25±557.64	*	4403.25±940.03	2797.1±988.12	0.35	−0.18	0.19	−0.24	0.39	0.85
		MT	1527±404.07	1852.5±612.62	*	2577.75±265.47	2324.65±760.73	0.33	0.30	0.19	−0.47	0.39	0.18
Early yield fruit number	eyfn	ML	12±2.45	8±1.63	*	14.5±2.38	14.06±4.27	0.30	−0.15	0.19	−0.24	0.39	0.83
		MT	10.25±1.71	7±2.16		11.75±1.26	13.13±3.79	0.29	0.66	0.19	0.41	0.39	0.68
Early yield av. fruit weight (gr)	eyfw	ML	147.12±16.2	343.78±23.15	*	301.98±18.43	195.3±36.17	0.19	0.29	0.19	−0.19	0.39	0.86
		MT	148.60±28.99	264.69±20.39	*	219.69±10.72	176.73±40.66	0.23	0.71	0.19	1.40	0.39	0.86
Fruit weight (gr)	fw	ML	153.92±32.04	392.75±70.51	*	383.5±65.76	252.33±56.48	0.23	0.02	0.19	0.20	0.39	0.84
		MT	180.42±20.54	294.75±44.27	*	304±38.46	214.65±40.8	0.19	−0.01	0.19	−0.13	0.39	0.88
Fruit length (cm)	fl	ML	21.83±2.98	9.88±0.43	*	17±0	14.15±1.87	0.13	0.38	0.19	0.38	0.39	0.91
		MT	20.33±5.57	8.17±1.46	*	11.08±1.97	11.46±1.65	0.15	0.44	0.19	0.96	0.39	0.74
Fruit diameter 1/2(cm)	fd1/2	ML	3.75±.031	10.25±0.63	*	7.75±0.21	6.70±0.81	0.14	0.12	0.19	−0.21	0.39	0.91
		MT	3.68±0.57	8.33±1.28	*	6.33±0.89	5.77±0.70	0.12	0.14	0.19	−0.19	0.39	0.70
Fruit diameter 3/4(cm)	fd3/4	ML	4.32±0.21	8.92±0.42	*	7.65±0.35	6.6±0.72	0.11	0.00	0.19	−0.22	0.39	0.88
		MT	4.45±0.40	7.6±1.36	*	6.13±0.81	5.84±0.63	0.11	0.11	0.19	−0.47	0.39	0.59
Fruit diameter max (cm)	fdmax	ML	4.40±0.21	10.52±0.75	*	8.05±0.35	7.05±0.85	0.12	0.13	0.19	−0.19	0.39	0.91
		MT	4.45±0.40	8.33±1.28	*	6.33±0.89	6.03±0.65	0.11	0.15	0.19	−0.34	0.39	0.60
Fruit shape	fs	ML	4.96±0.57	0.94±0.07	*	2.11±0.23	2.05±0.4	0.20	0.88	0.19	1.09	0.39	0.96
		MT	4.54±0.99	0.98±0.11	*	1.74±0.08	1.93±0.37	0.19	0.79	0.19	1.34	0.39	0.92
Peduncle length (cm)	pedl	ML	5.82±1.05	2.87±0.67	*	4.43±1.11	5.77±0.99	0.17	0.61	0.19	0.53	0.39	0.90
		MT	4.90±1.27	3.63±1.04		4.35±1.56	4.58±0.69	0.15	0.35	0.19	0.67	0.39	0.69
Fruit calix prickliness (0–3)	fcpri	ML	1.42±0.49	0.5±0	*	1.5±0.71	1.05±0.45	0.43	0.76	0.19	0.43	0.39	0.86
		MT	1.65±0.44	0.63±0.22	*	1.62±0.49	1.27±0.39	0.31	0.25	0.19	−0.21	0.39	0.64
Outer fruit firmness (kg/cm^2^)	outfir	ML	2.42±0.57	2.13±0.22		2.63±0.13	2.32±0.42	0.18	0.34	0.19	0.55	0.39	0.79
		MT	3.05±0.61	2.13±0.57	*	3.48±0.18	2.81±0.65	0.23	0.56	0.19	0.67	0.39	0.63
Number of locules	slon	ML	3.67±0.58	8±1.87	*	4±1.41	4.29±0.95	0.22	0.84	0.19	1.28	0.39	0.63
		MT	4.17±0.75	5.50±0.57	*	4.75±0.50	4.23±0.77	0.18	0.72	0.19	0.69	0.39	0.63
Flesh green ring (0–1)	gring	ML	1±0	0±0	*	1±0	0.66±0.47	0.72	−0.67	0.19	−1.57	0.39	0.98
		MT	1±0	0±0	*	0.88±0.25	0.61±0.44	0.72	−0.58	0.19	−1.56	0.39	0.98
Plant growth habit (1–3)	hab	ML	3±0	1±0	*	2±0	2.25±0.72	0.43	−0.46	0.19	−1.14	0.39	0.80
		MT	3±0	1±0	*	2±0	2.11±0.82	0.50	1.44	0.19	6.30	0.39	0.42
Leaf prickliness (0–3)	lepri	ML	2.83±0.29	0±0	*	0.5±0.1	0.09±0.17	0.38	1.70	0.19	1.67	0.39	0.71
		MT	3±0	0±0	*	0.5±0	0.13±0.2	0.27	1.37	0.19	0.72	0.39	0.64
N° of flower/inflorescence	flwin	ML	4±0	1±0	*	2±0	2.93±1.2	0.46	0.01	0.19	−0.61	0.39	0.33
		MT	5.5±0.71	1±0	*	2±0	3.06±1.51	0.31	0.55	0.19	−0.18	0.39	0.47

Significant mean differences between parental performance (Wilcoxon test) are indicated (*p<0.05), along with any skewness and kurtosis (with associated standard error (SE).

**Table 2:** Inter-trait Spearman correlations in the mapping population.

		ty	tyfn	tyfw	Ey	eyfn	eyfw	fw	fl	fd12	fd34	fdmax	fs	pedl	fcpri	outfir	slon	gring	hab	lepri	flwin
ty	ML	0.495*	0.852*	0.717*	0.943*	0.774*	0.689*	0.711*	0.433*	0.508*	0.607*	0.566*	−0.08	0.309*	−0.08	−0.204	0.196	0.08	0.340*	0.07	−0.02
	MT		0.885*	0.568*	0.864*	0.649*	0.539*	0.449*	0.451*	0.220*	0.323*	0.296*	0.15	0.14	0.01	0.02	0.13	0.02	0.06	0.04	0.08
tyfn	ML		0.532*	0.320*	0.816*	0.914*	0.314*	0.403*	0.492*	0.214*	0.328*	0.281*	0.13	0.251*	−0.200	−0.282*	0.05	−0.06	0.225*	0.10	0.187
	MT			0.190	0.776*	0.793*	0.249*	0.203	0.431*	0.00	0.12	0.10	0.267*	0.07	−0.13	0.03	−0.01	−0.08	0.08	−0.02	0.249*
tyfw	ML			0.592*	0.659*	0.272*	0.935*	0.856*	0.205	0.726*	0.778*	0.756*	−0.322*	0.309*	0.06	−0.03	0.313*	0.221*	0.387*	0.05	−0.289*
	MT				0.480*	0.02	0.788*	0.637*	0.196	0.516*	0.532*	0.526*	−0.162	0.10	0.272*	0.07	0.358*	0.12	−0.01	0.10	−0.220*
ey	ML				0.456*	0.843*	0.671*	0.710*	0.424*	0.495*	0.589*	0.554*	−0.07	0.295*	−0.11	−0.215*	0.175	0.00	0.315*	0.05	0.01
	MT					0.787*	0.541*	0.494*	0.425*	0.296*	0.415*	0.374*	0.10	0.14	−0.07	−0.02	0.10	−0.02	0.08	−0.08	0.04
eyfn	ML					0.487*	0.247*	0.368*	0.494*	0.178	0.279*	0.239*	0.15	0.252*	−0.182	−0.326*	0.02	−0.10	0.191	0.09	0.218*
	MT						−0.01	0.11	0.390*	−0.03	0.11	0.07	0.262*	0.06	−0.198	−0.05	−0.10	−0.12	0.07	−0.12	0.221*
eyfw	ML						0.569*	0.892*	0.13	0.778*	0.817*	0.806*	−0.391*	0.286*	0.00	0.01	0.290*	0.13	0.365*	0.02	−0.280*
	MT							0.688*	0.15	0.562*	0.578*	0.568*	−0.219*	0.10	0.15	0.09	0.318*	0.13	0.02	0.05	−0.166
fw	ML							0.897*	0.261*	0.819*	0.857*	0.848*	−0.342*	0.290*	0.02	−0.03	0.277*	0.09	0.325*	−0.02	−0.180
	MT								0.05	0.656*	0.678*	0.683*	−0.366*	0.06	−0.01	0.06	0.349*	0.05	0.09	−0.05	−0.200
fl	ML								0.602*	−0.205	−0.05	−0.12	0.750*	0.428*	−0.07	−0.196	−0.11	0.10	0.352*	0.14	0.162
	MT									−0.203	0.02	−0.07	0.783*	0.168	0.08	−0.03	−0.169	0.00	0.01	0.13	0.280*
fd1/2	ML									0.663*	0.943*	0.978*	−0.761*	0.09	0.00	0.00	0.359*	0.06	0.15	−0.09	−0.257*
	MT										0.912*	0.955*	−0.701*	−0.02	0.04	0.02	0.337*	−0.04	0.01	−0.12	−0.248*
fd34	ML										0.567*	0.983*	−0.650*	0.188	−0.01	−0.06	0.366*	0.09	0.203	−0.04	−0.236*
	MT											0.977*	−0.543*	0.01	0.01	0.01	0.269*	−0.07	0.01	−0.15	−0.200
fdmax	ML											0.611*	−0.712*	0.15	0.00	−0.04	0.352*	0.06	0.182	−0.07	−0.238*
	MT												−0.618*	0.01	0.03	0.01	0.297*	−0.07	0.00	−0.14	−0.214*
fs	ML												0.886*	0.199	−0.01	−0.09	−0.314*	0.04	0.14	0.13	0.233*
	MT													0.13	0.06	−0.02	−0.297*	0.02	−0.01	0.204	0.331*
pedl	ML													0.651*	0.03	−0.183	0.03	0.11	0.340*	0.238*	−0.14
	MT														0.05	−0.174	−0.01	0.04	−0.02	0.11	−0.01
fcpri	ML														0.541*	0.09	0.08	0.10	0.163	0.14	−0.05
	MT															−0.02	0.02	0.14	−0.12	0.355*	−0.10
outfir	ML															0.526*	0.01	−0.225*	−0.15	0.04	0.01
	MT																0.00	−0.291*	−0.09	0.12	0.168
slon	ML																0.285*	−0.01	−0.01	−0.07	−0.175
	MT																	0.08	0.10	−0.04	−0.169
gring	ML																	0.848*	0.306*	0.170	−0.06
	MT																		0.15	0.250*	−0.12
hab	ML																		0.567*	0.07	−0.212*
	MT																			−0.159	−0.04
lepri	ML																			0.465*	0.12
	MT																				0.08
flwin	ML																				0.477*
	MT																				

The values on the diagonal represent the inter-environment correlation for each single trait. Significant (p<0.05) correlations indicated by “*”.

### Identification of QTL Clusters

**Figure 2:**
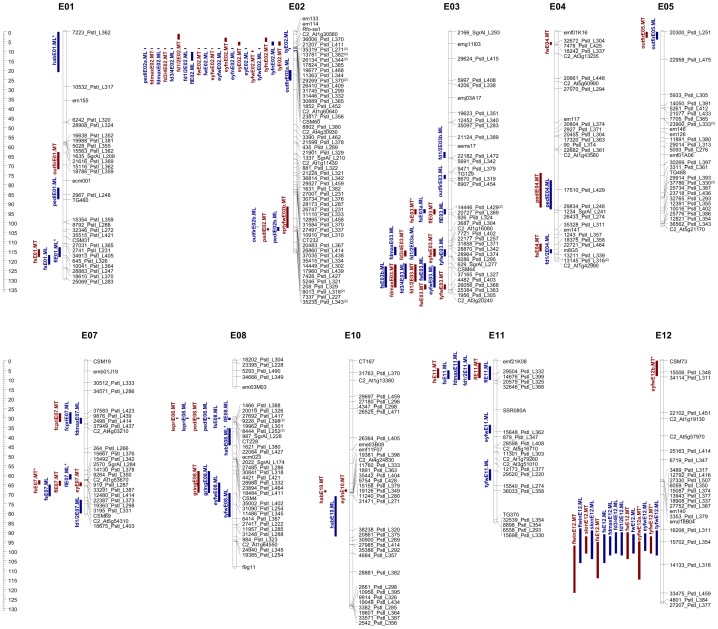
QTL location (only chromosomes harboring QTL are shown). The scale shown on the *left* indicates the chromosome length in cM. Marker names are shown to the *right*; the inclusion of a superscript near a marker name indicates the presence and number of additional co-localizing markers on the Barchi et al. (2012) map. Map positions of the QTL are given on the *left* of each chromosome. The length of the vertical bars represents the QTL confidence interval. QTL shown in *blue* were detected at ML, and those in *red* at MT. Epistatic QTL are prefixed by “*” where the QTL had already been detected by MapQTL software, and by “ep” where the QTL was newly detected.

In all, 105 QTL (of which 65 explained at least 10% of the phenotypic variance (PV), these are hereafter referred to as “major” QTL) were identified and mapped onto ten of the 12 eggplant chromosomes (Table 3), while no QTL were identified to E06 and E09. At ML, 62 QTL (33 major) were identified, while at MT, there were 43 QTL (32 major). Among the major QTL, 24 were expressed at both sites, eight were only detectable in ML and two only in MT; finally seven appeared as a major QTL in one of the sites but was retained as a minor one in the other. The genomic locations of these QTL are shown in Figure 2. Between one and four major QTL underlay the variation in each trait. Clustering of the QTL was common, and present on almost each chromosome. The high inter-trait correlations between some of the traits controlled by a cluster of QTL (Table 2) suggested that these clusters reflected either a set of closely linked loci or, more likely, a single pleiotropic locus. In the cluster on chromosome E02, the QTL were associated with early and total yield, as well as with several fruit traits (weight, length, diameters, peduncle length); the same region is known to harbor a major gene controlling resistance against *Fusarium oxysporum* [24]. The other major cluster was on chromosome E12, comprising QTL for eyfw and tyfw, fw, diameters, fs, slon and flwin. A smaller cluster was mapped to chromosome E01, comprising major QTL for fl and fs, although only the latter locus was expressed in both environments; the same chromosome also harbored other major QTL for hab and outfir (in MT). The two clusters present on chromosome E03 were associated with fruit diameters, fw, tyfw and eyfw, and the other to fl and fs, along with minor QTL for tyfw, fd1/2 and outfir. The chromosome E07 cluster involved fl, fs and ey, together with minor QTL for fruit diameters and a major one for fcpri. At the top of chromosome E08, one cluster of QTL determining lepri, fs fl and pedl was linked to a second one determining habit and effect on the green ring. Finally, the chromosome E11 cluster involved major QTL for fruit shape and diameters, and two minor QTL for tyfw and eyfw.

**Table 3:** QTL detected in the mapping population.

Trait code	Chr.	Montanaso Lombardo (ML)									Monsampolo del Tronto (MT)								
		GW	QTL	Position	Locus	CI	LOD	PV	A	D	GW	QTL	Position	Locus	CI	LOD	PV	A	D
ty	2	3.8	tyE02.ML	8.84	19677_PstI_L468	8.6–8.9	25.60	53.00	−2,856.6	2,079.2	3.9	tyE02.MT	5.91	At1g30580	5–7.1	9.16	23.70	−881.9	1,060.6
tyfn	2	3.9	tyfnE02.ML	5.91	At1g30580	5.1–7	24.78	51.90	−11.15	9.36	3.8	tyfnE02.MT	5.19	Rfo−sa1	3–5.2	9.46	24.60	−5.01	7.81
tyfw	2	3.8	tyfwE02.ML	8.84	19677_PstI_L468	8.6–8.9	20.38	31.10	−27.11	12.76	3.7	tyfwE02.MT	10.36	30889_PstI_L365	9.8–10.4	6.01	11.80	−12.49	−1.60
	3		tyfwE03.ML	117.54	9286_PstI_L295	114–117.6	5.27	6.30	−10.72	0.06		tyfwE03.MT	134.99	26056_PstI_L368	132.5–135	4.57	8.80	−9.72	1.13
	8		tyfwE08.ML	45.23	11486_PstI_L345	39.5–52.2	7.00	8.60	13.23	2.30									
	11		tyfwE11.ML	57.75	29520_PstI_L220	57–62.7	4.03	4.80	−9.28	−4.15									
	12		tyfwE12.ML	94.71	15702_PstI_L354	89.5–101.7	11.60	15.40	−18.00	−4.33		tyfw12.MT*	94.71	15702_PstI_L354	92–100.7	8.78	18.00	−14.48	−4.43
ey	2	3.8	eyE02.ML	8.84	19677_PstI_L468	8.6–8.9	24.24	51.3	−986,920	800.70	3.8	eyE02.MT	5.9	At1g30580	5.2–7	9.02	21.5	−353.63	563.67
	7											eyE07.MT	65.4	22387_PstI_L373	63.8–65.7	4.15	9.2	210.73	414.55
	2	3.7	eyfnE02.ML	8.84	19677_PstI_L468	8.6–8.9	20.65	45.6	−379.92	363.64	3.9	eyfnE02.MT	5.2	Rfo-sa1	3.1–5.2	7.66	17.7	−137.8	279.82
	10											eyfnE10.MT	68.58	11760_PstI_L333	67.5–68.6	5.48	12.1	153.17	134.80
eyfw	2	3.8	eyfwE02.ML	8.84	19677_PstI_L468	8.6–8.9	23.17	35.9	−327.46	174.76	3.8	eyfwE02.MT	8.63	17824_PstI_L385	8.5–8.7	6.13	12.8	−224.54	254.48
	3		eyfwE03.ML	132.57	4482_PstI_L403	123–134	5.38	6.3	−115.45	409.66		eyfwE03.MT	117.54	9286_PstI_L295	113.7–117.8	3.80	7.6	−146.26	407.13
	8		eyfwE08.ML	39.58	31090_PstI_L254	34–44	4.69	5.4	123.83	−496.39									
	11		eyfwE11.ML	37.20	15648_PstI_L362	33.6–38	3.99	4.6	−107.40	−544.24									
	12		eyfwE12.ML	94.71	15702_PstI_L354	90.5–99.7	9.29	11.5	−176.53	0.299		eyfwE12a.MT	106.73	14133_PstI_L316	94.7–114.7	4.33	8.7	−160.41	654.38
	12											eyfwE12b.MT	5.94	15006_PstI_L348	0–8	3.88	7.8	570.73	−204.84
fw	2	3.7	fwE02.ML	8.84	19677_PstI_L468	8.6–8.8	21.16	40.00	−53.87	27.88	3.9	fwE02.MT	8.84	19677_PstI_L468	8.6–8.9	22.00	34.70	−36.50	17.48
	3		fwE03.ML	123.42	37165_PstI_L327	121.4–130.4	4.28	6.20	−14.53	16.76		fwE03.MT	134.99	26056_PstI_L368	132.5–135	9.82	12.80	−19.89	3.61
	4											fwE04.MT	6.33	7478_PstI_L425	5.3–6.7	4.54	5.40	11.35	11.80
	12		fwE12.ML	94.71	15702_PstI_L354	91–101	6.51	9.80	−25.81	2.92		fwE12.MT	94.71	15702_PstI_L354	89.5–103.7	4.86	5.90	−14.26	3.31
fl	1	3.8	flE01.ML*	117.24	645_PstI_L326	113–118	8.78	12.00	0.95	0.28	3.7								
	2		flE02.ML	13.86	C2_At1g60640	10.5–14.8	8.00	10.80	–0.66	1.04									
	3		flE03.ML	94.67	20727_PstI_L369	92.9–95.6	12.37	17.80	1.16	0.64		flE03.MT	94.67	20727_PstI_L369	93–95.7	6.30	11.50	0.82	0.42
	7		flE07.ML*	62.16	14130_PstI_L378	59.5–62.2	4.10	5.20	0.65	0.22		flE07.MT	65.4	22387_PstI_L373	63–65.5	5.98	10.90	0.81	0.38
	8		flE08.ML	0.61	20019_PstI_L326	0–1	5.06	6.50	0.70	0.06									
	11		flE11.ML	6.9	29504_PstI_L332	3.0–10	7.55	10.10	0.90	0.11		flE11.MT	6.9	29504_PstI_L332	1–8.9	8.56	16.20	1.01	−0.38
fd1/2	2	3.8	fd1/2E02.ML	8.84	19677_PstI_L468	8.6–8.9	17.24	21.70	–0.64	0.33	3.8	fd1/2E02.MT	1.12	SSR114	1–4.1	4.79	10.20	−0.33	0.12
	3		fd1/2E03a.ML	117.54	9286_PstI_L295	113–117.5	6.54	7.00	–0.33	0.04		fd1/2E03.MT	123.42	37165_PstI_L327	122–131.4	10.30	23.90	−0.45	0.01
	3		fd1/2E03b.ML	65.97	22182_PstI_L472	63–66	4.35	4.30	−0.30	−0.21									
	4		fd1/2E04.ML	115.98	M8G5	114–116	4.37	4.30	−0.27	0.06									
	7		fd1/2E07.ML	74.87	At5g54310	73.4–80	4.92	5.30	−0.32	0.03									
	11		fd1/2E11.ML	6.9	29504_PstI_L332	2.1–10	9.82	10.90	−0.47	0.11									
	12		fd1/2E12.ML	94.71	15702_PstI_L354	90.5–102	8.26	9.00	−0.39	−0.17									
fd3/4	2	3.8	fd3/4E02.ML	8.84	19677_PstI_L468	8.6–8.9	20.26	38.20	−0.69	0.32	3.8	fd3/4E02.MT	8.84	19677_PstI_L468	8.6–8.9	5.82	12.80	−0.33	0.20
	3		fd3/4E03.ML	123.42	37165_PstI_L327	121.5–129.5	5.63	8.40	–0.25	0.19		fd3/4E03.MT	117.54	9286_PstI_L295	114.7–117.6	7.74	17.50	−0.34	−0.08
	12		fd3/4E12.ML	94.71	15702_PstI_L354	90.5–100.7	5.42	8.10	−0.30	−0.05									
fdmax	2	3.7	fdmaxE02.ML	8.84	19677_PstI_L468	8.6–8.9	19.42	30.20	−0.73	0.33	4.0	fdmaxE02.MT	8.84	19677_PstI_L468	8.6–8.9	6.08	13.10	−0.34	0.20
	3		fdmaxE03.ML	113.69	28964_PstI_L374	112.7–117	7.22	9.30	−0.37	−0.01		fdmaxE03.MT	123.42	37165_PstI_L327	122–132.4	8.70	19.50	−0.37	−0.05
	7		fdmaxE7.ML	32.9	2498_PstI_L414	30–33	3.73	4.50	−0.23	−0.23									
	11		fdmaxE11.ML	6.9	29504_PstI_L332	0–10	6.12	7.70	−0.37	0.10									
	12		fdmaxE12.ML	94.71	15702_PstI_L354	92.5–102	8.43	11.00	−0.40	−0.21									
fs	1	4.0	frsE01.ML	118.3	10041_PstI_L364	117–122	9.79	9.40	0.18	0.01	3.9	fsE01.MT	117.24	645_PstI_L326	114–118	5.69	6.40	0.14	0.01
	3		fsE03a.ML	94.67	20727_PstI_L369	93–95	15.60	16.30	0.25	0.14		fsE03.MT*	94.67	20727_PstI_L369	93–95.7	20.00	28.20	0.29	0.14
	3		fsE03b.ML	132.58	4482_PstI_L403	123–134	4.55	4.00	0.12	0.01									
	4											fsE04.MT	114.14	22721_PstI_L464	111–115	4.23	4.70	0.07	−0.12
	7		fsE07.ML	65.4	22387_PstI_L373	65–71	8.70	8.20	0.18	0.07		fsE07.MT*	62.81	12480_PstI_L414	62.7–64.8	11.30	14.00	0.21	−0.01
	8		fsE08.ML	0	1466_PstI_L388	0–0.5	7.03	6.50	0.16	−0.07									
	11		fsE11.ML	6.9	29504_PstI_L332	5–9.5	17.80	19.50	0.28	−0.01		fsE11.MT	6.9	29504_PstI_L332	4–8.9	15.10	19.80	0.25	−0.06
	12		fsE12.ML	94.71	15702_PstI_L354	91–106	7.15	6.70	0.12	0.13		fsE12.MT	106.73	14133_PstI_L316	95.7–113.7	4.50	5.00	0.09	0.13
pedl	1	3.9	pedlE01.ML	85.28	2967_PstI_L248	81.7–87.7	6.85	9.70	0.42	–0.13	3.7								
	2		pedlE02a.ML	8.84	19677_PstI_L468	8.6–8.9	7.84	11.30	–0.40	0.51									
	2		pedlE02b.ML	103.27	14449_PstI_L302	102.3–104.3	7.24	10.30	−0.41	−0.31		pedlE02.MT	104.33	17960_PstI_L439	103–104–35	7.77	15.10	−0.36	−0.22
	4		pedlE04.ML	82.59	17510_PstI_L429	77.8–93	9.94	14.80	0.57	−0.06		pedlE04.MT	82.59	17510_PstI_L429	74–92	8.59	17.10	0.43	−0.02
	8		pedlE08.ML	0	1466_PstI_L388	0–0.5	5.27	7.30	0.40	0.04		pedlE08.MT	0.61	20019_PstI_L326	0–0.6	4.24	7.90	0.27	0.07
fcpri	7	3.9	fcpriE07.ML	30.08	9876_PstI_L439	27–33	4.25	11.80	0.21	0.16	4.3	fcpriE07.MT	30.08	9876_PstI_L439	27.5–32	4.79	12.80	0.23	0.14
outfir	1	3.8									4.0	outfirE01.MT	69.55	18786_PstI_L359	63–72	5.17	12.00	0.34	0.12
	2		outfirE02a.ML	22.45	CSM60	19.99–25.45	7.34	14.60	202.00	−310.69									
	2		outfirE02b.ML	102.31	37035_PstI_L438	102.2–102.3	4.34	8.10	164.65	9.25									
	3		outfirE03.ML	76.97	8670_PstI_L319	75–77	4.31	7.80	−173.50	8.03									
	5		outfirE05.ML	0	20300_PstI_L251	0–4	7.80	15.00	221.60	−132.02		outfirE05.MT	0	20300_PstI_L251	0–3	7.03	16.80	0.39	−0.07

**Trait code**	**Chr.**	**Montanaso Lombardo (ML)**									**Monsampolo del Tronto (MT)**								
		**GW**	**QTL**	**Position**	**Locus**	**CI**	**LOD**	**PV**	**A**	**D**	**GW**	**QTL**	**Position**	**Locus**	**CI**	**LOD**	**PV**	**A**	**D**
slon	12	3.8	slonE12.ML	94.71	15702_PstI_L354	89–104	5.77	15.70	−0.48	−0.30	3.9	slonE12.MT	94.71	15702_PstI_L354	92–100.7	9.24	23.90	−0.52	−0.13
gring	8	4.0	gringE08.ML	29.19	35002_PstI_L402	28–39.3	93.63	93.70	0.49	0.47	4.0	gringE08.MT	29.19	35002_PstI_L402	28–39.2	75.50	89.20	0.45	0.41
hab	1	3.9	habE01.ML*	0	7223_PstI_L362	0–21	4.96	11.00	0.33	0.13	3.7								
	8		habE08.ML*	7.35	22084_PstI_L427	5.5–14.4	4.65	10.30	0.33	0.00									
	10		habE10.ML	89.07	38238_PstI_L320	71.3–92	5.16	11.50	−0.34	0.13		habE10.MT	68.92	1891_PstI_L363	68–69	5.37	14.20	−0.37	0.22
lepri	8	3.1	lepriE08.ML	0	1466_PstI_L388	0–0.5	5.95	16.20	0.10	–0.04	4.4	lepriE08.MT	0	1466_PstI_L388	0–0.5	5.34	14.60	0.11	−0.04
flwin	12	3.8	flwinE12.ML	94.71	15702_PstI_L354	88.5–106.5	6.21	16.80	0.66	0.42	3.8	flwinE12.MT	106.73	14133_PstI_L316	97–121.7	6.82	18.20	0.86	0.13

For each trait the genome-wide threshold (GW) at p = 0.05 (as determined from 1,000 permutations) is indicated. The marker mapping closest to each QTL and which parental allele contributed positively to the trait are indicated, along with its confidence interval (CI), the estimated LODs at the QTL peak (LOD), the PV explained and the additive (A)/dominance (D) contribution. Asterisks indicate QTL showing epistatic interactions.

### QTL Determining Agronomic Traits in Eggplant

All the QTL detected in the mapping population, their statistics and associated markers are reported in Table 3.

#### Traits related to total (ty, tyfn, tyfw) and early (ey, eyfn, eyfw) yield

A particularly large effect ty QTL (*tyE02*) explained 53.0% of the PV in ML (23.7% of the PV in MT), and mapped to the same E02 region (8.8 cM) as major QTL for tyfw (*tyfwE02.ML*, responsible for 31.0% of the PV) ey (*eyE02.ML*) and eyfn (*eyfnE02*.*ML*), as well as *fwE02* and *eyfwE02* both of which were expressed at both sites. The confidence interval (CI) associated with all of these QTL was just 0.3 cM. In MT, *tyE02* mapped to position 5.9 cM with a CI of 5.0–7.1 cM, overlapping with *tyfnE02.ML, eyE02.MT* and *eyfnE02.MT*. A further major QTL for tyfw (also expressed at both sites) mapped on E12 at 94.7 cM, underlying the marker 15702_PstI_L354, and explaining 15.4% of the PV in ML and 18.0% in MT. Three additional tyfw QTL were detected on E03, E08 and E11; *tyfwE03* and *tyfwE08* were both coincident with an fd QTL. Apart from *tyfwE08*.*ML,* all the positive alleles for traits related to total yield (ty, tyfn and tyfw) derived from ‘67/3’.

In ML, the unique major QTL associated to ey trait (*eyE02*) explained 24.4% of the PV and mapped to E02 at 8.8 cM within the major yield-related traits QTL cluster. In MT, the same QTL explained 21.5% of the PV and was located at 5.9 cM. A minor, but in this case MT-specific, ey QTL (*eyE07*.*MT*) was located on E07, explaining 9.2% of the PV. Positive alleles at some ey QTL were inherited from ‘67/3’ (*eyE02*), but others from ‘305E40’ (*eyE07*.*MT*). The major eyfn QTL (*eyfnE02*) co-localized with *tyfnE02.ML*; in ML it explained 45.6% of the PV, and in MT 17.7%. The positive allele was inherited from ‘67/3’. A second, MT-specific, eyfn QTL mapped to E10 (*eynfE10*.*MT*), explained 12.1% of the PV and had inherited the positive allele from ‘305E40’. A major eyfw QTL mapping to E02 at 8.8 cM (*eyfwE02*) explained 23.2% of the PV at ML and 12.8% at MT. Its location coincided with that of the fw, ey and ty QTL described above. A second eyfw locus was detected on E12 at 94.7 cM, linked to the marker 15702_*Pst*I_L354, and explaining 11.5% of the PV in ML, but less than 10% in MT. Additional minor eyfw QTL mapped to E03 (both sites), E08 and E11 (ML only) and E12 (only in MT). The loci *eyfwE03* and *eyfwE08.ML* clustered with QTL controlling fd and tyfw. With the exception of *eyfwE08*.*ML*, the positive alleles were all inherited from ‘67/3’.

#### Fruit weight (fw)

Three fw QTL were mapped in ML and four in MT. The major QTL *fwE02* was expressed at both sites, and explained 40.0% of the PV in ML and 34.7% in MT. Its location coincided with ty, ey and a fd QTL. The *fwE03* locus explained 12.8% of the PV in MT, but <10% in ML. The remaining two QTL were both minor; *fwE12* was expressed at both sites but *fwE04.MT* was specific for MT. With the exception of *fwE04*.*MT*, the positive alleles were all derived from ‘67/3’.

#### Fruit length (fl)

Six fl QTL were detected in ML, distributed over E01, E02, E03, E07, E08 and E11. The two largest effect loci *flE03* and *flE11* explained respectively 17.8% and 10.1% of the PV, and were detected at both sites; *flE01.ML* and *flE02.ML*, although of equivalent effect, were ML-specific. *flE07* was a minor QTL in ML, but explained 10.0% of the PV in MT, while the minor locus *flE08.ML* was ML-specific. With the exception of *flE02*.*ML*, all the positive alleles were derived from ‘305E40’.

#### Fruit diameter (fd1/2, fd3/4, fdmax)

The three fd parameters were highly inter-correlated (Table 2). Considered separately, in ML three to seven QTL were mapped to E02, E03, E04, E07, E11 and E12. While in MT a major QTL for each fd trait was located on each of E02 and E03. In ML, the set of QTL having the largest effect on fd were *fd1*/*2E02*, *fd3*/*4E02* and *fdmaxE02*, explaining, respectively 21.7%, 38.2% and 30.2% of the PV. The *fd3*/*4E02* and *fdmaxE02* loci were also detected as major QTL in MT. The segment containing these E02 loci also influenced fw, ty, ey, tyfw and pedl. The *fd1/2E03*, *fd3/4E03* and *fdmax.E03* loci had a less marked effect in ML than did the E02 ones, and were also detected in MT, where they explained, respectively, 23.9%, 17.5% and 19.5% of the PV. For all the detected QTL, positive alleles were contributed by ‘67/3’.

#### Fruit shape (fs)

Four fs QTL were detected in ML, mapping to E01, E03 (two loci) and E07; three of these were also expressed in MT. The two loci *fsE03b.ML* and *fsE04MT* were site-specific and minor. The locus *fsE03a* explained 16.3% of the PV in ML and 28.2% in MT. The positive alleles at each QTL were inherited from ‘305E40’.

#### Peduncle length (pedl)

Five pedl QTL were mapped in ML (E01, E02 (two loci), E04 and E08). Although the proportions of PV explained and the additive effects were approximately the same for all of them, the decreasing alleles from E02 was inherited from ‘67/3’, while the others derived from ‘305E40’. Three of the loci (*pedlE02b*, *pedlE04* and *pedlE08*) were also confirmed in MT.

#### Fruit calyx prickliness (fcpri)

*fcpriE07* mapped at 30.1 cM, and was expressed in both sites (responsible for 11.8% of the PV in ML and 12.8% in MT). The locus was linked to the marker (9876_*Pst*I_L439) and the allele for increased prickliness derived from ‘305E40’.

#### Resistance to mechanical penetration (outfir)

Four outfir QTL were detected in ML, mapping to E02 (*outfirE02a/b*), E03 and E05. o*utfirE02a.ML* and *outfirE05* were both major QTL and explained, respectively, 14.6% and 15.0% of the PV. The latter locus was also expressed in MT (16.8% of the PV). An additional major locus (12.0% of the PV) on E01 was detected only in MT. With the exception of *outfirE02b* and *outfirE03*, all the positive alleles were inherited from ‘305E40’.

#### Number of seed locules (slon)

A single major QTL was detected on E12 in both environments at 94.7 cM. It explained 15.7% of the PV at ML and 23.9% at MT. The ‘67/3’ allele was associated with an increased number of locules.

#### Green ring (gring)

A single major QTL (*gringE08*) was identified for the presence of the green ring in the flesh. It was linked to the marker 35002_*Pst*I_L402, and explained nearly all of the PV at both sites (93.7% at ML, 89.2% at MT). The ‘305E40’ allele was associated with the green ring’s presence.

#### Plant growth habit (hab)

Three major hab QTL, all explaining a similar proportion of the PV and all associated with similar additive effects, were located on E01, E08 and E10 exclusively in ML. The only major effect QTL detected in MT mapped to E10 and explained 14.2% of the PV. All prostrate habit associated QTL alleles were inherited from ‘67/3’.

#### Leaf prickliness (lepri)

The single lepri major QTL *lepriE08* was expressed in both sites, where it explained 16.2% (ML) and 14.6% (MT) of the PV. As for fcpri, the positive allele was derived from ‘305E40’.

#### Number of flowers per inflorescence (flwin)

The single QTL *flwinE12* explained 16.8% of the PV at ML and 18.2% at MT. The allele from ‘305E40’ was associated with a greater number of flowers per inflorescence.

### Epistasis

Epistatic interactions were evaluated by considering the two sites as independent replicates (Table 4, Figure 2). In ML, epistatic interactions were observed for fl and hab. For the former trait, a pair of previously detected QTL (flE01.ML* and flE07.ML*) displayed a significant level of additive x dominant epistasis, with an individual variance of 1.3%. Meanwhile, for hab, habE01.ML* and habE08.ML* both displayed significant additive x additive interaction with an individual variance of 3.2%. In MT, epistatic interactions were observed for fs and tyfw. For fs, the two QTL fsE03.MT* and fsE07.MT* both displayed significant dominance x dominance epistasis, with an individual variance of 2.3%. For tyfw, the already identified QTL tyfwE12.MT*, together with a *de novo* QTL (*eptyfwE02b.MT*) displayed a significant degree of additive x additive epistasis. The combined site analysis showed that none of the additive effect x site or dominance effect x site interactions were statistically significant at p<0.05.

**Table 4:** Epistatic effects detected at p<0.05.

**Trait**	**QTL_i**	**position_i**	**range_i**	**QTL_j**	**position_j**	**range_j**	**AA**	**PV(AA) %**	**AD**	**PV(AD) %**	**DA**	**PV(DA) %**	**DD**	**PV(DD) %**
fl	flE01.ML*	119.3	117.2–129.1	flE07.ML*	69.7	63.8–73.4			0.81	1.28				
														
														
fs	fsE03.MT*	88.7	81.7–96.7	fsE07.MT*	64.8	62.8–72.7							−0.29	2.26
														
hab	habE01.ML*	12.0	0.0–24.0	habE08.ML*	10.4	0.0–14.5	−0.34	3.19						
														
														
tyfw	eptyfwE02b.MT	33.4	29.4–34.5	tyfwE12.MT*	95.7	91.6–102.7	12.70	3.62						

AD, DA DD: additive x dominant, dominant x additive, dominant x dominant interactions, respectively. PV(AD), PV (DA) and PV (DD)%: the contribution of, respectively, the AD, DA and DD interaction.

### Candidate Gene Identification based on Orthology with Tomato

**Figure 3:**
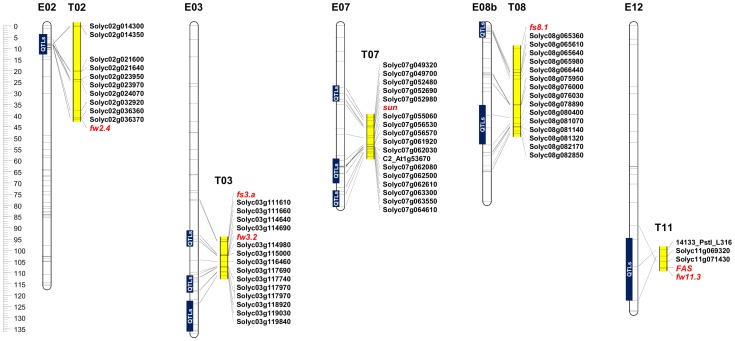
Synteny between eggplant chromosomes E2, E3, E7, E8b and E12 and parts of tomato chromosomes T2, T3, T7, T8 and T11. The physical locations of the tomato genes *FW2.4*, *fs3*.*a*, *fw3*.*2*, *sun*, *fs8.1*, *FAS* and *fw11*.*3* are shown in italics and in red. ‘QTL’ shown in a blue box indicate a cluster of eggplant yield, fruit dimension and weight QTL. The scale on the *left* indicates the length of eggplant chromosomes in cM, while distances on the tomato chromosome segments derived from their physical position on the genome [54].

The tomato fruit weight QTL *fw2.4* [6] lies in a region which is syntenic to a part of eggplant E02 where several fruit dimension QTL proved to be clustered (Figure 3). Similarly, the location of tomato *fw3.2* [13] corresponds to the E03 region harbouring fruit weight and diameters QTL, while *flE03* and *fsE03* may well be orthologous to the tomato *fs3.a* locus described by Grandillo *et al*. [6] The region harbouring *sun* in tomato, a gene required for the production of an elongated fruit [25], [26] is syntenic to the chromosome E07 region harbouring fl and fs QTL. This is the same region identified as carrying the eggplant fruit shape QTL *fs7.1* [16] and QTL involved in fruit set [17]. The tomato fruit shape QTL *fs8.1* [3] region is represented in eggplant by an E08 region harbouring fl and fs QTL, at least expressed in ML. The tomato fruit weight associated genes FASCIATED (*FAS*) and *fw11.3* [14] lie on a part of T11 syntenic with a segment of E12 harbouring QTL controlling diameters, fw, fs, slon and tyfw.

Search for other candidates of the eggplant QTLs was conducted by analyzing the tomato genes included in the syntenic region defined by the CI of each QTL. The green ring locus on E08 is marked by 35002_*Pst*I_L402, the sequence of which is similar to that of tomato *Solyc08g077050*, encoding for a ferredoxin family protein. In *Arabidopsis thaliana* this protein is a component of Photosystem I chlorophyll production [27]. A similar analysis of the markers included in *fcpriE07* CI identified three potential candidate sequences, namely *Solyc07g049700*.1 (encoding a disease resistance protein), *Solyc07g051820* (encoding cellulose synthase), *Solyc07g045290* (encoding a long chain fatty acid-CoA ligase involved in the suberin pathway). For *lepriE08*, possible candidate genes identified were *Solyc08g005120*.2 (encoding a cinnamoyl-CoA reductase-like protein involved in the lignin pathway), *Solyc08g005170*.2 (encoding a heat stress transcription factor) or *Solyc08g005280* (encoding a cellulose synthase-like protein). For peduncle length, a search in the region harbouring *pedlE02b* identified *Solyc02g088690* (encoding a UDP-glucose 6-dehydrogenase involved in the formation of hemicellulose and pectin), *Solyc02g089130* (encoding a COBRA-like protein, which has a major role in the cell wall synthesis) and *Solyc02g089640* (encoding a cellulose synthase-like C1–2 glycosyltransferase family 2 protein). Finally, for the slon and flwin QTL on E12, the possible candidates identified in the CI were *Solyc11g068620* and *Solyc11g068750* (both encoding NAC domain proteins).

## Discussion

### Phenotyping, QTL Mapping and Clustering of Agronomic Trait Loci

Increasing the weight of the fruit, improving its shape, and minimizing prickliness have provided the focus of much of the selection pressure applied to eggplant in the process of its domestication [17]. Although fruit size in cultivated types can vary by at least an order of magnitude (from 20–30g to 600–700g), total yield tends nevertheless to be correlated with the number of fruits produced by each plant. In a previous study heterosis for total yield was detected in ten eggplant hybrids obtained by crossing germplasm accessions, which was comparable to the one detected in commercial hybrids. However no significant heterosis was observed for some quality traits as well as fruit weight, thus yield was mainly attributed to the increase in fruit set [28]. Our results, together with those obtained previously, are of particular interest to address future eggplant breeding programs designed at selecting high yielding genotypes.

Prickliness is an important quality trait, as during handling the prickles can damage the skin of the fruit or even harm the personnel involved in harvest and post-harvest operations [29]. Despite this, types with a very prickly calyx are preferred in some regions, like Nagpur (India), on the basis of its perceived association with better organoleptic quality. Fruit firmness is important for storage purposes, while an upright plant habit is beneficial as it eases harvesting. The number of flowers formed per inflorescence is clearly correlated to the number of fruit set and therefore to yield potential. In most cultivars, this number lies in the range 1–3, but can reach 9–10 in some forms. However, since the largest fruits develop from the most important flower, a common, but costly practice is the manual removal of secondary flowers (or the primary flower in the case of cluster types cultivation). The abundance of seeds locules has significance because the presence of seeds within the fruit reduces its commercial value. Finally, the presence and thickness of the green ring inside the skin is regarded as a negative trait by most consumers because it gives the impression that the fruit is still unripe.

Although the major quality traits are well recognized by eggplant breeders, few attempts have been made to date to elucidate their genetic basis. The earliest investigations described QTL for fruit shape and pigmentation [16], while Frary et al. [18] exploited an interspecific F_2_ population to identify QTL underlying 18 morphological traits. Cross species comparisons within the Solanaceae have suggested that 12 of these QTL have probable orthologues in at least one of the species. Doganlar et al. [17] focused on the inheritance of various fruit traits and anthocyanin pigmentation, while most recently, Barchi et al. [21], using the same F_2_ population as here, located QTL associated with anthocyanin content and identified syntenic relationships between the eggplant and tomato genomes. A common feature of many QTL studies is that phenotype interacts with the environment, producing QTL x environment interactions which are difficult for the breeder to handle. However these were not evident in the present study, and most of the traits proved to be highly heritable (Tables 1 and 2). Clustering of fruit-related QTL was commonplace, reflecting linkage and/or (more likely) pleiotropy; for example alleles influencing fl or fd are naturally likely to and also affect fs, while alleles influencing fl and fd can also be expected to influence fw. Four chromosomal regions (on E02, E03, E11 and E12) appear to harbour the major QTL underlying fruit dimension, size and yield. A particularly important region is the E02 segment between 5 and 10 cM, which therefore represents an excellent target for developing markers for yield.

The number of fruit produced per plant is a key component of yield potential. In this population, a single major tyfn QTL expressed at both sites was uncovered and the same chromosomal region also harboured an eyfn QTL, at least at MT. At ML the QTL position was shifted by 3–4 cM to a region containing major QTL underlying ty and tyfw at ML, as well as fw and several other fruit-related traits at both sites. The co-location of these QTL offers an attractive molecular breeding opportunity. The substantial positive correlation between ey and ty implies furthermore that any selection pressure applied on yield at the first few harvests will apply a similar pressure on overall productivity; this would allow for a marked reduction in the labour and cost required for yield selection, given that the crop produces fruit over a prolonged period.?QTL underlying related traits have a proven tendency to co-localize [17], and the present experiment produced plenty of examples of this tendency. Thus, for example, the E02 and E03 QTL controlling seven yield-related traits were all clustered, as were the QTL determining three different fruit shape-related traits on E07 and E11, and those controlling four fruit shape-related traits on E12. The E07 and E11 clusters are very likely the same loci as those identified by Doganlar et al [17]. In contrast, the lepri and fcpri QTL were scattered over two chromosomes, and there were also examples of linkage between QTL controlling quite unrelated traits (for example *flE08.ML*, *fsE08.ML*, *pedlE08* and *lepriE08*, and *flwinE12* with various E12 fruit dimensions and weight loci). An unanticipated linkage between anthocyanin content and prickliness QTL was also encountered by Doganlar et al [17], who concluded that negative selection imposed on leaf prickliness may also have affected loci controlling pedl, fl and fs, while selection for fs acted simultaneously on flwin. While Doganlar et al. [17] defined a major QTL located on chromosome E06 controlling the prickliness of the leaf, stem, petiole and fruit calyx, in the present population control of these characters mapped to locations on E07 and E08, and there was little correlation between fcpri and lepri, presumably resulting from the different parental lines used to generate the F_2_ population. Doganlar et al [17] used an interspecific map while an intraspecific one was employed in the present work; maybe the prickliness genes of the wild species used by Doganlar et al. [17] are not the same than those of our population. In fact, when crossing two cultivated non-prickly species (e.g., *S. melongena* with S. *aethiopicum* or *S. macrocarpon*) the interspecific hybrid is frequently prickly [30] **,** which suggests that different (recessive) genes are present in each species conferring absence of prickles). From a breeding point of view selection for reduced prickliness in the fruit calyx cannot indirectly be performed by an early selection for absence/low prickles in the leaves; in addition, markers for both the traits are needed to apply MAS for these features.

Collard et al. [31] have suggested that a QTL can be classified as major only if it explains at least 10% of the PV, although a more nuanced definition also requires a demonstration of stable expression over time and space [32]–[34]. On the latter basis, of the 62 QTL expressed in ML and the 43 in MT, at least one per trait was a major locus. The LOD score associated with the least convincing of these was just over 4 (*fcpriE07*) while the most convincing was >90 (*gringE08*); the PV explained varied from ∼10% (*flE11*) to ∼94% (*gringE08*). The stability of most of these QTL is particularly promising in terms of their exploitation in the context of marker-assisted selection. Some of the major fruit dimension QTL (e.g., *flE07*, *fd1/2E03* and *fd3/4E03*) explained quite a divergent proportion of the PV in the two environments, which presumably reflects the consequence of the different growing conditions at the two sites. A number of the minor QTL, as classified on the basis of the proportion of the PV explained (e.g. *fwE12*, *tyfwE03*, *fsE12* and *pedlE08*), were stably expressed, while others were site-specific (e.g. *fwE04.MT*, *fd1/2E04.ML*). This phenomenon is a commonplace of QTL related to yield in a number of different species [35].

### Parental Alleles, Transgressive Segregation and Epistasis

In the majority of cases, the parental origin of the QTL alleles reflected the performance of the parents; thus, for example, most of the positive alleles at fw, fd and ty were inherited from ‘67/3’, while those at fl, fs, fcpri, lepri, hab and flwin were derived from ‘305E40’. Transgressive segregation arises where a progeny of a cross has inherited a non-parental combination of alleles acting towards the same direction [36]. The transgressive progeny with respect to pedl in ML fitted this model, as they carried two segments carrying a positive QTL allele, one inherited from each parent. However, the model failed with respect to many of the traits (ty, tyfn, tyfw, eyfn, outfir, fcpri and slon in ML, and ty, ey, fw, tyfn, outfir, slon and hab in MT); this was taken to imply that some (minor) QTL still remain to be identified.

A number of environmental-specific examples of epistasis were identified, although none of these explained a substantial proportion of the PV (1.3–3.6%). Presumably the analysis of data generated from both sites hampered the detection of epistatic interactions, an effect explainable by invoking interference from other QTL in the background [35]. Overall, the lack of epistasis (it only affected four of the 20 traits) is a positive outcome as it greatly simplifies the exploitation of the QTL in a breeding context. Although the analysis carried out with QTLNetwork 2.1 [37] on the combined data set produced no significant QTL x Environment interactions, some identified QTL with the MQM approach were location-specific: for this reason we cannot rule out the presence of QTL x Environment interaction.

### Synteny and Putative Orthologous QTL

The genetic basis of fruit weight and dimension has been widely explored in the Solanaceae, especially in tomato and sweet pepper [6], [8], [10], [13], [14], [23], [38]. Extensive synteny do exist between the tomato and eggplant genomes, thereby allowing genetic inferences to be made in eggplant based on the much greater knowledge for the tomato genome [21], [39], [40]. Specifically, the gene content of an eggplant genomic region harbouring a particular QTL can be assumed to be similar to that in the orthologous segment of the tomato genome. Examples of this are provided firstly by the chromosome T02 gene/QTL *fw2.4* identified by Grandillo et al. [6] in the context of the yield-related QTL located here to eggplant chromosome E02; and secondly the T03 region harbouring *fw3.2* and *fs3.a* [6], [13] in relation to the E03 QTL underlying fruit weight, dimension and yield.

In tomato, fruit shape is under the joint control of *ovate* on chromosome T02, *sun* (T07) and *fs8.1* (T08). The former gene encodes a protein which negatively regulates plant growth [41], while *sun* of is only effective in post fertilization [25], [26]. The QTL *fs8.1* is responsible for the slightly elongated shape of processing tomatoes [5]. In the present eggplant population, fruit shape QTL were identified in the regions orthologous to those harboring *sun* and *fs8.1*, but not *ovate.* Among other genes involved in the determination of tomato fruit weight/shape, *FAS*, which encodes a transcription factor controlling locule number and thereby fruit mass [42], is tightly linked to the fruit weight QTL *fw11.3* [14]; this location suggests possible orthology with the E12 fl, fd, fs, tyfw QTL cluster. On the other hand, no eggplant equivalent of either *LOCULE NUMBER* [43] or *fw2.2* [18] were evident. Using a different mapping population, however, Doganlar et al. [17] were able to identify a possible orthologue of *fw2.2.* The failure in the present case to do so may well reflect the lineage of the parental line ‘305E40’, which is known to carry a segment derived from *S. aethiopicum,* including the *Rfo-sa1* locus conferring resistance to the soil-borne *Fusarium oxysporum* f. sp. *melongenae,* and located in the distal portion of its chromosome E02 [24]. The marker genotype in this chromosome region is identical to that of the *S. aethiopicum* progenitor from position 0 cM (locus em133) to position 10.4 cM (30889_*Pst*I_l365) (Table S1).

The tomato genome annotation also allowed for a presumptive identification of a candidate gene for the green ring locus *gringE08*, namely a member of the ferredoxin gene family. Ferrodoxins are involved in chlorophyll synthesis, and the green pigment is known to be chlorophyll. Association between this tomato locus and the expression of the green ring in eggplant flesh may be gathered through a deep functional analysis of the cloned gene(s) underlining the QTL together with a biochemical characterization of the composition of the flesh. A similar analysis of the *fcpriE07* QTL identified as possible candidates genes encoding a cellulose synthase, a long chain fatty acid-CoA ligase 3, a cinnamoyl-CoA reductase-like protein and a cellulose synthase-like protein. All of these proteins are components of the cellulose, lignin and suberin production pathways, required to form prickles. A possible, but less plausible candidate genes were encoding either a disease resistance protein or a heat stress transcription factor, which may chime with the idea that prickliness is an expression of the response to stress, and in particular represents a means of reducing the plant’s palatability to herbivores [29]. The potential candidate genes for the pedl QTL included encoders of either a UDP-glucose 6-dehydrogenase, a COBRA-like protein or a cellulose synthase-like C1–2 glycosyltransferase family 2 protein. All these gene products are connected with cell wall synthesis and thus to peduncle elongation. Finally, a potential candidate gene for the *flwin* and *slon* QTL was an encoder of a NAC domain protein. NAC domain proteins are involved in the formation of the shoot apical meristem, various floral organs and lateral shoots, in plant hormonal control and in the stress response [44], therefore fulfill functions coherent with the *flwin* and *slon* QTL.

## Conclusions

We have demonstrated here the utility of the combination of a densely populated genetic map and an appropriate segregating intraspecific population for elucidating the genetic basis of breeder’s traits in eggplant. Major QTL were identified for yield and its components, as well as for fruit dimension, shape and firmness, the number of seed locules present, the length of the peduncle, prickliness and growth habit. A feature of the analysis was the presence of a number of QTL clusters. The robustness of many of these major QTL offers the possibility of exploiting them via marker assisted selection. Finally, it was possible to demonstrate that a comparative genetic approach relying on the much larger tomato knowledge base can help to identify potential candidate genes, which provide an additional genomic resource relevant for marker assisted selection and for further synteny studies in the Solanaceae.

## Methods

### Permission

No specific permits were required for the described field studies, which took place in two experimental fields at the CRA-ORL in Montanaso Lombardo and CRA-ORA in Monsampolo del Tronto (Italy). These field plots were used by the authors of this paper affiliated to the aforementioned institution (LT, NA, NF, FF, VB and GLR) for phenotypic characterization of the eggplant mapping population.

### Mapping Population and the Evaluation of Phenotype

A population of 156 F_2_ plants, previously obtained by crossing the eggplant lines ‘305E40’ and ‘67/3’ [21], [23], was employed. The double haploid female parent ‘305E40’ possesses the resistance locus to the soil-borne fungus *Fusarium oxysporum* f. sp. *melongenae Rfo-sa1* [24]. This eggplant genotype was derived from an interspecific somatic hybrid *Solanum aethiopicum* gr. *gilo*(+)*S. melongena* cv. Dourga [22], which underwent several cycles of backcross with recurrent eggplant genotypes (lines DR2, and Tal1/1) prior to selfing and anther culture. The ‘67/3’ line is an F_8_ selection from the intra-specific cross cv. ‘Purpura’ x cv. ‘CIN2’.

The mapping population was grown, along with both parents and the F_1_ hybrid, in the field at two sites, namely ML (Montanaso Lombardo 45°20′N, 9°26′E) and MT (Monsampolo del Tronto 42°53′N; 13°47′E) in 2009. Each F_2_ individual was replicated by establishing vegetative cuttings. At both sites, the material was arranged as a set of two randomized complete blocks with two replicate plants per entry per block. The 20 traits scored are detailed in Table 1, and were measured in the fashion defined by IBPGR [45] and the ECPGR eggplant descriptors [46]. Twelve weekly fruit harvests were made starting in mid July and lasting until early October. The number of fruits harvested per plant (tyfn) and their mean weight (tyfw) allowed for the calculation of total yield (ty). The first five harvests were combined to give early yield (ey), number of early fruit (eyfn) and mean early fruit weight (eyfw). Two representative fruits per plant picked between the first and fourth harvests were chosen to characterize fruit weight (fw), fruit length (fl), the diameter sampled in three parts of the fruit (fd1/2, fd3/4 and fdmax), peduncle length (pedl), fruit shape (fs) (the ratio between fl and fdmax), calyx prickliness (fcpri) (scored on a zero (no prickles) to three (many strong prickles) scale). Resistance to mechanical penetration (outfir) was measured by inserting a manual penetrometer halfway between the peduncle and the distal end of the fruit. The fruit was cut transversely in the seed region to ascertain the number of seed locules present (slon) and the presence/absence of a green ring (gring) inside the skin. Whole plant traits were measured prior to the first harvest; these comprised growth habit (hab), scored on a scale from one (prostrate) to three (upright), leaf prickliness (lepri) (scored in the same way as for calyx prickliness) and the number of flowers per inflorescence (flwin), estimated from a count of the flowers present in five inflorescences.

### Statistical Analyses and QTL Detection

Statistical analyses were performed using R software [47]. A conventional analysis of variance was applied to estimate genotype and environment effects based on the linear model *Y_i_*_j_ = *μ*+*g*_i_+*b*_j_+*e*_ij_, where *μ*, *g*, *b* and *e* represent, respectively, the overall mean, the genotypic effect, the block effect and the error. Broad-sense heritability values were given by σ^2^_G_/([σ^2^_G_+σ^2^_E_]/n), where σ^2^_G_ represented the genetic variance, σ^2^_E_ the residual variance and n the number of blocks. Correlations between traits were estimated using the Spearman coefficient, and normality, kurtosis and skewness were assessed with the Shapiro-Wilks test (α = 0.05). Segregation was considered as transgressive when at least one F_2_ individual recorded a trait value higher or lower by at least two standard deviations than the higher or lower scoring parental line. QTL detection was based on the Barchi et al. [21] map, constituted of 415 markers (339 SNPs, 2 HRMs, 3 CAPSs, 11 RFLPs, 33 SSRs and 27 COSII) and spanning 1,390 cM. Putative QTL location was determined by both interval [48] and MQM [49]–[51] mapping, as implemented in MapQTL v5 software [52]. QTL were initially identified using interval mapping, after which one linked marker per putative QTL was treated as a co-factor in the approximate multiple QTL model. Co-factor selection and MQM analysis were repeated until no new QTL could be identified. LOD thresholds for declaring a QTL to be significant at the 5% genome-wide probability level were established empirically by applying 1,000 permutations per trait [53]. Additive and dominance genetic effects, as well as the percentage of the PV explained by each QTL were obtained from the final multiple QTL model. The program QTLNetwork 2.1 [37] was used to analyse each set of environment’s data separately to identify epistasis, and was then extended across both environments to identify any QTL x environment interactions present. QTL effects were estimated on the basis of the Markov Chain Monte Carlo (MCMC) method. A type I error level of 0.05 was applied. The genome scan employed a 10 cM window and a 1 cM walk speed. Critical F values were obtained by 1,000 permutations and a threshold of 0.05 was applied to assign significance to a QTL or to an epistatic effect. Individual QTL were prefixed by a trait abbreviation, followed by the relevant chromosome designation, and were suffixed “a” or “b” where more than one QTL mapped to a single linkage group; ML or MT was added as a suffix where the QTL was expressed in a site-specific manner. Epistatic effects were indicated by adding “*” to the label of a major established QTL, while “ep” was added to a newly detected QTL. MapChart v2.1 software [54] was draw the resulting maps. Syntenic regions of the genome tomato (sequence build 2.40; [55]) were accessed to identify candidate genes co-localizing with the eggplant QTL. Initial searches were conducted using 20-kb sections and, for sections of interest, additional searches were performed using 10 kb sections. Putative tomato orthologs of the eggplant genes were identified by Blast search in the tomato gene indices at DFCI [56].

## Supporting Information

Figure S1 The distribution of phenotype over the mapping population for each trait at each site. Parental (‘305E40’, ‘67/3’) and the F_1_ hybrid (‘F_1_’) performance indicated by arrows.
(PDF)

Table S1 Haplotype variation within the 0–10.4 cM region of chromosome E02, showing the presence of a *Solanum aethiopicum* chromosome segment in ‘305E40’.
(XLSX)
