# Supplementary figures and images for: QTL Mapping in Eggplant Reveals Clusters of Yield-Related Loci and Orthology with the Tomato Genome
Source: PLoS One. 2014 Feb 21;9(2):e89499. doi: 10.1371/journal.pone.0089499 (PMC3931786; doi:10.1371/journal.pone.0089499)

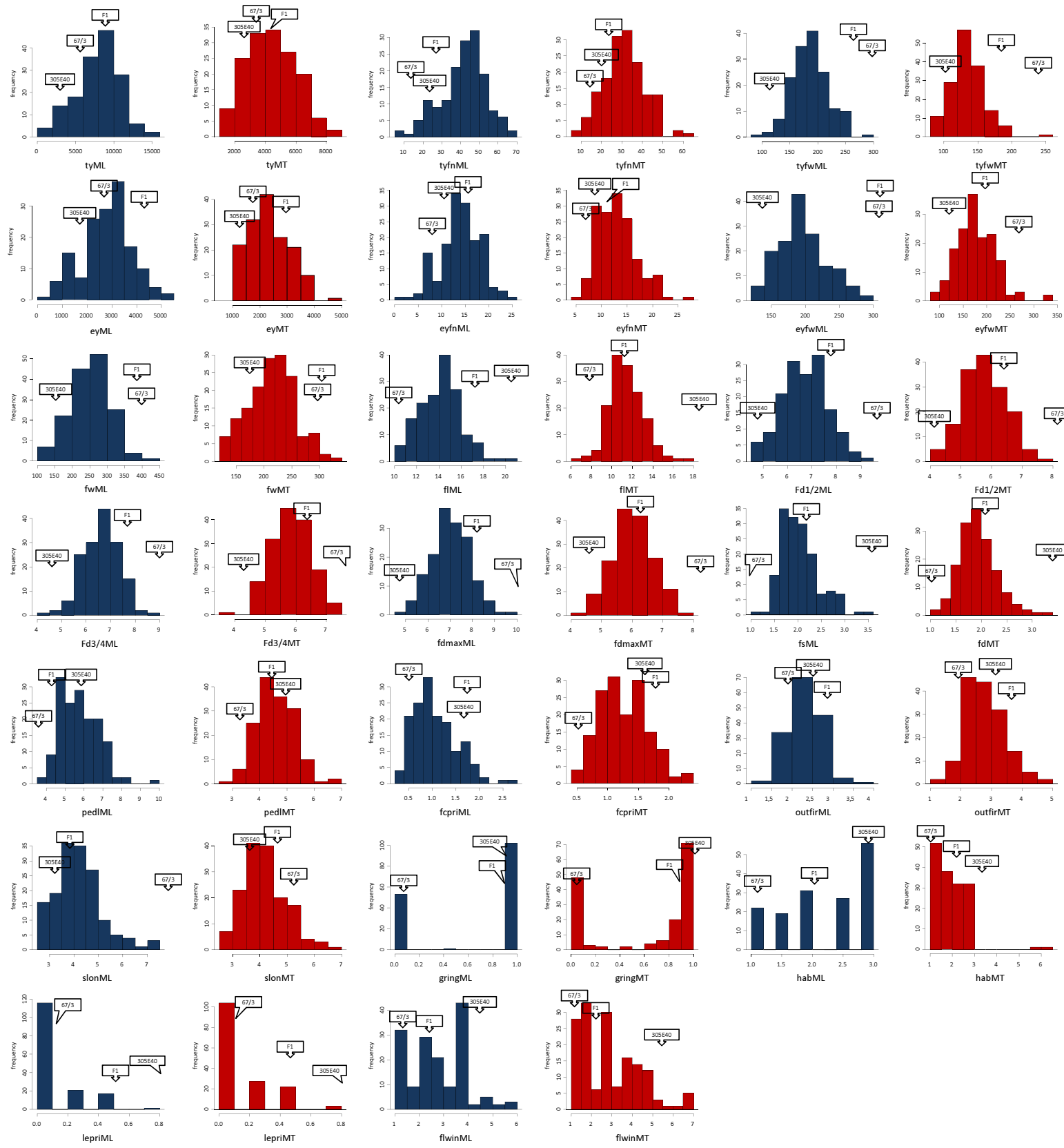

Supplement: Figure S1 — The distribution of phenotype over the mapping population for each trait at each site. Parental (‘305E40’, ‘67/3’) and the F1 hybrid (‘F1’) performance indicated by arrows. (PDF) [file pone.0089499.s001.pdf]
